# Peritoneal dialysis related eosinophilic peritonitis: a case report and review of the literature

**DOI:** 10.1186/s12882-022-03027-8

**Published:** 2023-01-12

**Authors:** Zhang Qingyan, Xia Yangyang, Zhang Miao, Jiang Chunming

**Affiliations:** Department of Nephrology, Nanjing University Medical School Affiliated Nanjing Drum Tower Hospital, 321 Zhongshan Road, 210008 Nanjing, China

**Keywords:** Eosinophilic peritonitis, Peritoneal dialysis, Peritonitis, Infection, Case report

## Abstract

**Background:**

Overt eosinophilic peritonitis (EP) is a relatively uncommon complication of peritoneal dialysis (PD), although not rare. Here we reported a case of EP relieved after changing dialysate.

**Case presentation:**

A 28-year old male patient developed cloudy PD effluents within the first month after PD started. Cytological study of PD effluents showed elevated white blood cells and polynuclear cells. Bacteria culture of PD effluents repeated for several times were all negative, and no pathogen was found by metagenomics next generation sequencing (mNGS). Antibiotic therapy for 28-day was ineffective. Based on these and increased eosinophils in peritoneal fluid, he was finally diagnosed as EP. PD dialysate was changed (consists of the same buffer agent and electrolytes, but is packed in bags that do not contain PVC), and the patient’s PD effluent became clear. Of note, EP did not relapse 5 months later when the patient started to use the former PD solution again.

**Conclusion:**

Although PD effluent turbidity almost always represents infectious peritonitis, there are other differential diagnoses including EP. For patients with cloudy fluid accompanied by mild symptoms who do not response to antibiotic therapy, it is reasonable to consider the possibility of this disease. EP tends to heal spontaneously, however, antihistamines or glucocorticoids are required sometimes to avoid catheter obstruction. For patients with no obvious incentives, replacement of dialysate may be useful.

## Background

Eosinophilic peritonitis (EP) was first reported in 1967 [1], which is usually a non-infectious peritoneal dialysis (PD) related peritonitis. The incidence of EP varies in the literature because of different diagnostic criteria [2–5], but generally showed a tendency to decrease. However, with the increased utilization of PD, the total number of patients with EP may still elevate. EP is often misdiagnosed as infective peritonitis because the two diseases have similar clinical symptoms and often overlap, which leads to the overuse of antibiotics, sometimes even results in serious complications and poor prognosis. Here we reported a case of EP manifested as cloudy PD effluent and relieved after changing peritoneal dialysate.

## Case presentation

A 28-year old male patient developed end-stage kidney disease due to chronic glomerulonephritis and a double-cuff straight Tenckhoff peritoneal dialysis (PD) catheter was inserted on February 1, 2021. The patient received daytime ambulatory peritoneal dialysis (DAPD) since then. His PD regime included 2,000 ml of 1.5% PD solution (Dianeal 1.5%, Baxter, International Inc.) with a total dialysis dose of 8 L per day. His daily PD ultrafiltration volume was about 400 ml and urine output about 1,500 ml/24 h.

From February 18, 2021, the patient observed turbid PD effluent without abdominal pain, fever or decline of PD ultrafiltration volume. Dialysate effluent was obtained for laboratory evaluation including cytological study and bacterial culture. The cytological study showed a white blood cell (WBC) count of 1419 × 10^6^/L, with 56.7% polynuclear cells, while the effluent culture was negative. He was diagnosed as PD-related peritonitis and received empirical antibiotic treatment by intraperitoneal injection of teicoplanine combined with third-generation cephalosporin and oral fluconazole for 14 days. The PD effluent was still cloudy. Other than that, the patient stated no discomfort. The effluent culture was repeated 5 days after the antibiotic application, but still negative. On March 2, 2021, the patient developed fever, and a dialysate effluent cytological study was repeated, which showed a WBC count of 2665 × 10^6/L, with 73.0% polynuclear cells. A complete blood count (CBC) was also performed this time which showed a WBC count of 6.5 × 10^9/L, with 65.3% neutrophils and 15.0% eosinophils. The patient was hospitalized on March 4, 2021.

The patient had a history of renal hypertension for 2 years. Irbesartan combined with amlodipine has been used since PD started and his blood pressure was well controlled. The patient also had anemia of CKD and Chronic Kidney Disease–Mineral and Bone Disorder (CKD-MBD). Home medications also included roxadustat, ferrous succinate and calcitriol. He had no history of tuberculosis or close contact with tuberculosis patients. He did not smoke, had no history of alcohol use, allergy and no previous surgeries except PD catheter insertion.

Physical examination on admission showed a body temperature of 38.5℃, a blood pressure of 113/80 mmHg, and a heart rate of 82 bpm. There was no purpura or rash. Pulmonary and cardiac auscultation was normal. The abdominal examination revealed no tenderness, guarding or rebound pain on palpation. There was no edema in both legs.

**Fig. 1:**
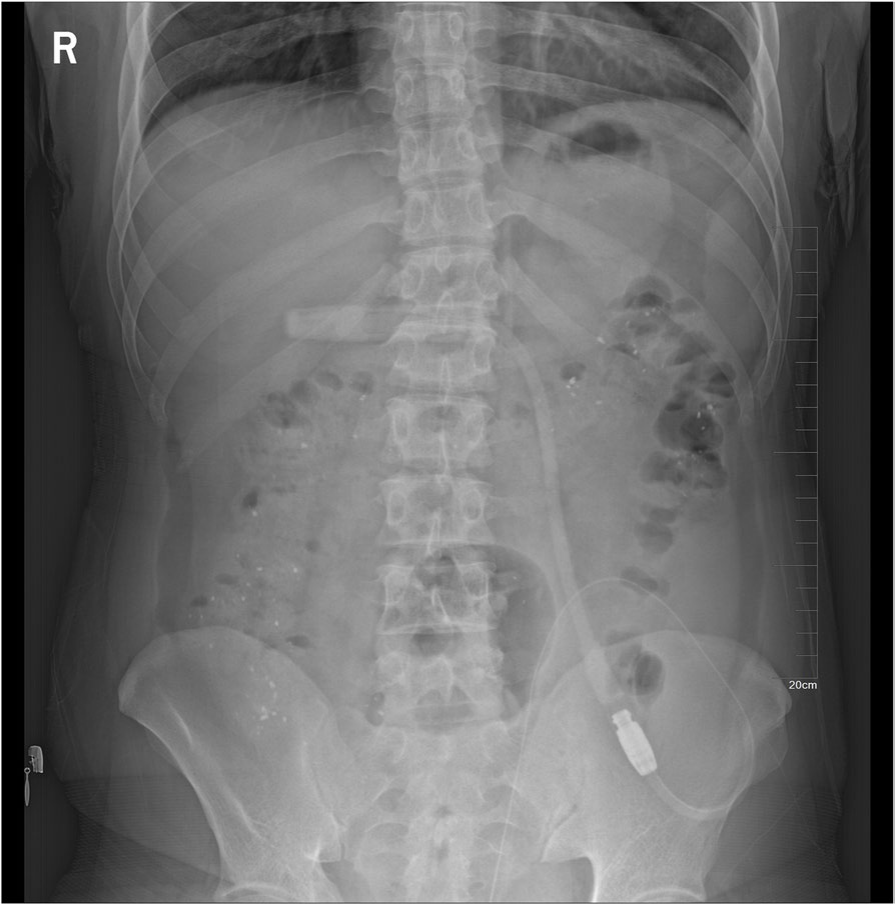
Abdominal X-ray of the patient

Results of laboratory tests and imaging study after admission are as follows: Cytological study of dialysate effluent showed a WBC count of 796.0 × 10^6/L, with 68.3% polynuclear cells. CBC showed WBC count 6.4 × 10^9/L (with 65.5% neutrophils and 14.9% eosinophils ), hemoglobin 87 g/L, and platelet count 220 × 10^9/L. Biochemical analysis revealed normal alanine aminotransferase and aspartate aminotransferase. Serum albumin was 32.4 g/L, and serum creatinine was 741 umol/L. His blood procalcitonin (1.5ng/ml) and C-reactive protein (CRP) (88.3 mg/l) were both high. Serum IgE was within normal range (60 IU/ml). During hospitalization, bacterial culture, Gram staining and acid fast bacilli staining of peritoneal dialysis effluents were all negative. Blood culture, detection of tuberculosis infected T cells, and tuberculosis antibody were negative as well. The G test of blood invasive fungi was 4.4pg/ml, and the GM test of blood invasive fungi was 0.07, both within normal range. Abdominal X-ray and CT scan did not show intra-abdominal free gas (Fig. 1).

**Fig. 2:**
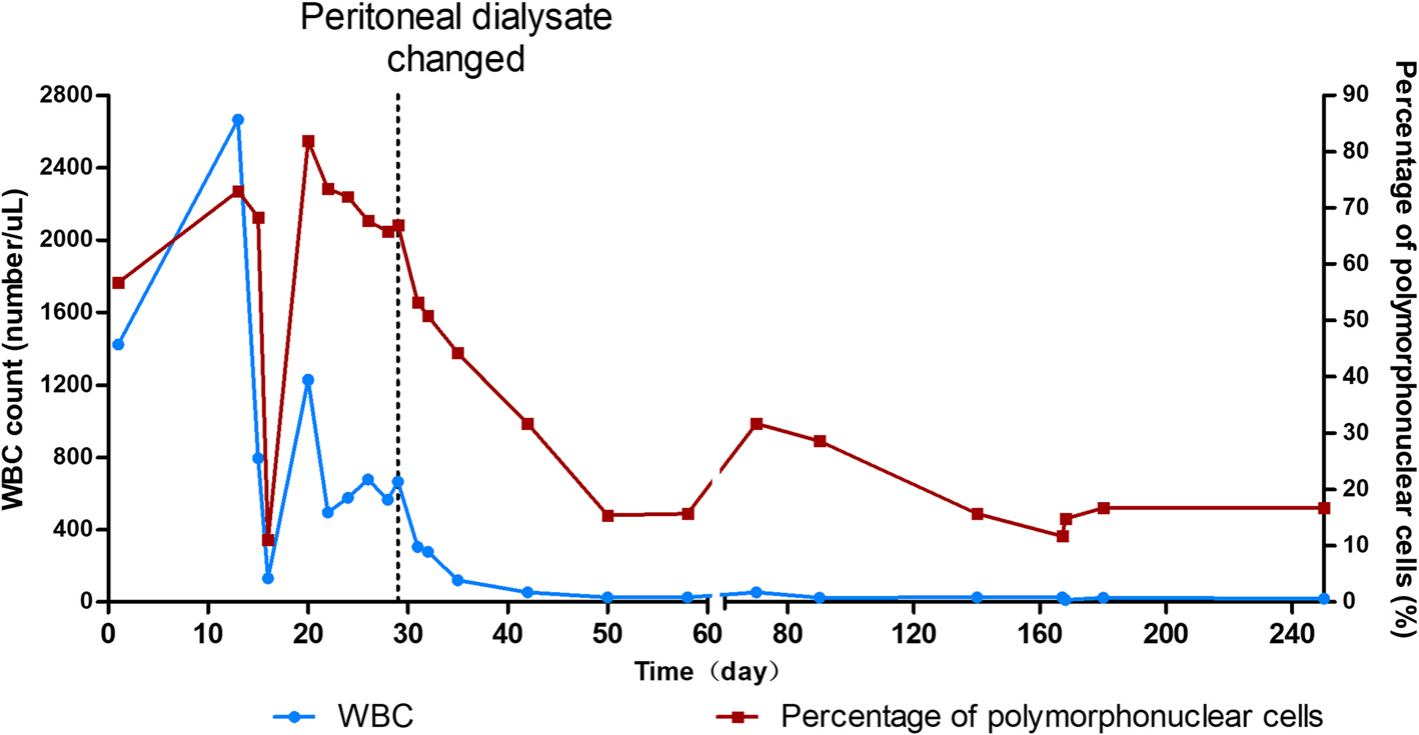
Total WBC count and percentage of polymorphonuclear cells in PD effluent

**Fig. 3:**
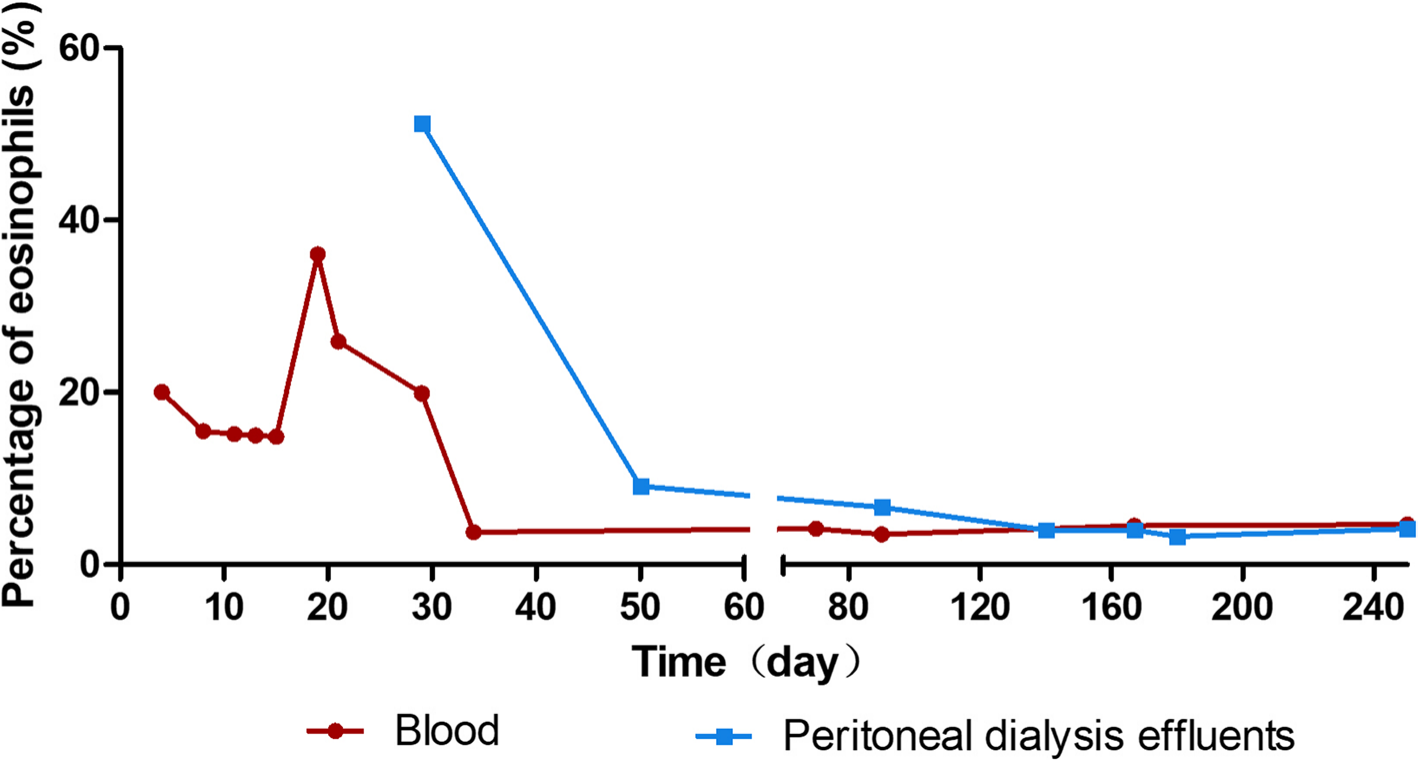
Percentage of eosinophils in blood and peritoneal dialysis effluent

**Fig. 4:**
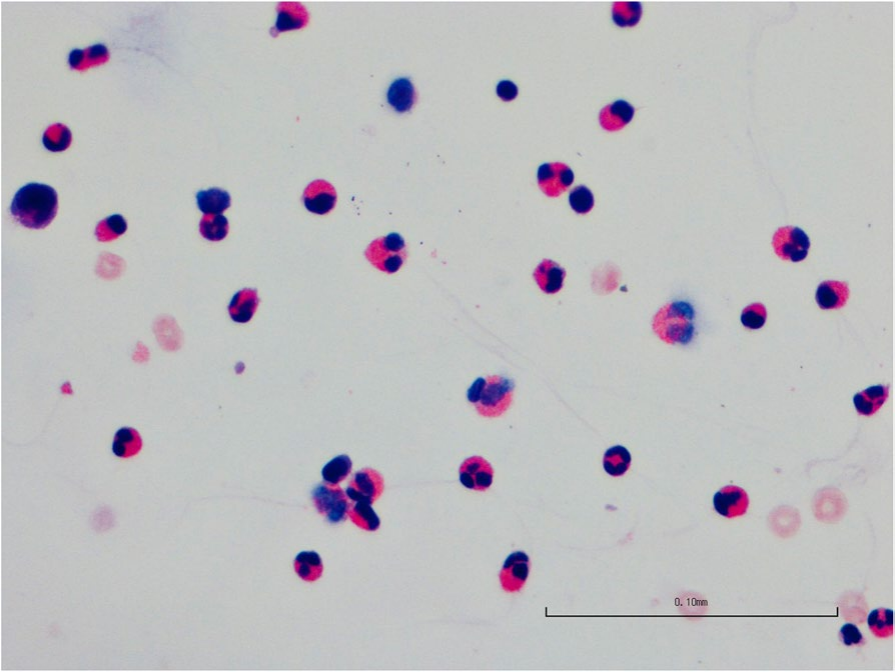
Eosinophilic cells in PD effluent (HE, collected on an Olympus BX51 equipped with a 40x objective)

Since the patient had fever and elevated blood eosinophils, dexamethasone (5 mg) was prescribed for one time, meanwhile, antibiotic therapy was continued, including intraperitoneal injection of teicoplanine and aminoglycosides (amikacin). The patient’s temperature returned to normal and his dialysis effluent went clear with WBC count of 131 × 10^6/L on March 5, 2021. Unfortunately, he observed turbid dialysate effluent again 4 days later. Still he denied abdominal pain or other discomfort. PD effluent cytological study showed a WBC count of 1228 × 10^6/L, with 81.9% polynuclear cells. Amikacin was replaced by imipenem/cliastatin and teicoplanine as well as fluconazol continued (antibiotics used during peritonitis are summarized in Table 1). However, the cloudy PD effluents did not improve, and the cytological study of effluents still showed elevated WBC counts (Fig. 2). Meanwhile, his blood CRP and procalcitonin decreased to normal, bacteria culture of PD effluents repeated for several times were all negative, and no pathogen was found by metagenomics next generation sequencing (mNGS). Considering that the proportion of blood eosinophils increased significantly during hospitalization (Fig. 3), we asked the laboratory to report the eosinophilic proportion of PD effluent (usually only the counts and proportion of polynuclear cells were reported), which showed that the proportion of eosinophils was as high as 71.2%, and exfoliated cells in PD effluent demonstrated an elevated eosinophils (Fig. 4). The patient was diagnosed as PD related EP, and antibiotics and fluconazol were discontinued. PD dialysate was changed to Daorun PD fluid (Wuhu Daorun Pharmaceutical Co., Ltd), which consists of the same buffer agent and electrolytes as Baxter fluid (Table 2), but is packed in bags that do not contain PVC, to see whether EP was caused by material of PD bags. The patient’s PD effluent went clear and the WBC counts of PD effluents decreased gradually (Fig. 2). The eosinophils in blood and PD effluents decreased as well (Fig. 3).

**Table 1:** Antibiotics used during peritonitis

Time	Dosage regimen
Day 1 to 14	Teicoplanin 0.2 g + Ceftazidime 1 g IP qd, Fluconazol 100 mg po qd
Day 15 to 20	Teicoplanin 0.2 g + Amikacin 0.2 g ip qd, Fluconazol 100 mg po qd
Day 21 to 28	Teicoplanin 0.2 g ip qd, Imipenem/Cliastatin 500 mg ip bid, Fluconazol 100 mg po qd

**Table 2:** Composition of Daorun peritoneal dialysate (G-1.5%)

C_6_H_12_O_6_·H_2_O	NaCl	C_3_H_5_NaO_3_	CaCl_2_·2H_2_O	MgCl_2_·6H_2_O	Osmotic pressure	PH
1.5 g/100ml	0.538 g/100ml	0.448 g/100ml	0.0183 g/100ml	0.0051 g/100ml	344mosmol/l	4.5 ~ 6.5

Since the new PD dialysate was not covered by his medical insurance, the patient decided to use Baxter’s PD solution again on August 2, 2021. His PD effluents were clear and cytological study of dialysate effluent showed normal WBC counts and proportions of polymorphonuclear cells and eosinophils during follow-up (Figs. 2 and 3).

## Discussion and conclusions

EP is defined as the presence of more than 100 eosinophils/mm^3^ of PD effluent or as an eosinophil count > 10% of the total nonerythrocyte count [6]. This entity tends to occur shortly after PD is started, although it can develop as late as 1 ~ 2 years or more after dialysis [3, 7]. Our patient developed EP within the first month of the initiation of PD, which is consistent with that reported in the literature.

The main clinical presentation of EP is cloudy peritoneal dialysis fluid that is unresponsive to antibiotics. Usually, as in this case, other symptoms of peritonitis including abdominal pain, tenderness and rebound tenderness are mild or even absent, and dialysate culture is negative, which is helpful in its distinction from bacterial peritonitis [6]. However, as microbial-induced peritonitis is the most common cause of PD effluent turbidity, clinicians may still make an incorrect diagnosis of infectious peritonitis, which leads to the overuse of antibiotics and may result in adverse events and extra costs. Therefore, it is of importance that clinicians be aware of the possibility of EP in patients with asymptomatic fluid turbidity or patients with cloudy PD effluent but mild clinical symptoms.

The exact pathophysiological mechanisms or causes of EP are still not fully understood. In addition to increased eosinophils in PD effluent, it has been shown that 32 ~ 57% of patients with EP have elevated peripheral blood eosinophils as well [7–9]. Chan et al. [2] reported a higher mean serum IgE concentration in patients who experienced peritoneal eosinophilia than in those without. Our patient also showed significantly elevated peripheral blood eosinophils, although his serum IgE level was normal. These together strongly support the suggestion that EP is an allergic reaction that is not localized to the surface of the peritoneum. Several mechanisms have been suggested to cause this allergic reaction, including presence of air in the peritoneal cavity [10, 11], effect of dialysis solutions or dialysate additives such as antibiotics [9, 12, 13] and streptokinase [14], and even effect of oral drugs [15]. However, none of these could well explain the situation in our patient. Solary et al. [16] reported a case of EP caused by plasticizers in PD bags, whose cloudy dialysate disappeared after substituting glass bottles for plastic containers. However, what substances could be involved was unknown. Chan et al. [17]. reported a case of severe EP relieved by changing from Dianeal PD fluid (packed in bags made of PVC) to Stay-Safe Balance solution (Fresenius) (a low glucose degradation product solution at neutral PH). However, it is unclear whether the resolution of EP was due to the properties of the Stay-Safe Balance PD fluid or whether it was due to the difference in their materials used for the plastic container bags, as all components of the packaging for Stay-Safe Balance solution are made of PVC-free Biofne. Our patient’s EP relieved after changing to PD fluid containing the same buffer agent and electrolytes but packed in bags that do not contain PVC. Meanwhile, the peritoneal and blood eosinophils dropped to normal. Since hemodialysis using PVC tubing has been linked with blood eosinophilia [18], it is possible that PVC used as plasticizers can be released from PD bags under certain conditions, and cause EP in certain cases. However, since the number of eosinophils in dialysate was calculated rather late in this patient, it cannot be completely excluded that EP was caused by other factors such as antibiotics given for peritonitis, and that at the beginning cloudy dialysate was due to mild culture-negative infectious peritonitis.

EP often remits spontaneously, however, in some cases it may last for months or even recurrent, and antihistamine drugs or glucocorticoids are required, in case that the cloudy PD effluent block the PD tube. Our patient’s cloudy PD effluent had a transient improvement after intravenous injection of dexamethasone, suggesting that steroids are effective. However, it is still controversial how long they should be used. Some suggest that glucocorticoids should be used continuously for more than 4 weeks to reduce disease recurrence [7]. Meanwhile, in some cases, EP relieved promptly after steroids prescription and there was no recurrence following quick drug withdrawal. Our patient only received one single dose of dexamethasone and suffered relapse of cloudy PD effluent, but achieved sustained relief after changing dialysate. Of note, EP did not relapse 5 months later when the patient started to use Baxter’s PD solution again, which reminds us that transient replacement of dialysate may be useful for EP patients without drugs, infection, air entering the abdominal cavity or other obvious incentives.

Although PD effluent turbidity almost always represents microbial-induced peritonitis, there are other differential diagnoses including EP [19], which often occurs in the early stage of PD and is usually a benign disorder but unresponsive to routine antimicrobial therapy. For patients with cloudy fluid accompanied by mild symptoms who do not response to antibiotic therapy, it is reasonable to consider the possibility of EP [20]. EP tends to heal spontaneously, however, antihistamines or glucocorticoids are required sometimes. For patients with no obvious incentives, replacement of dialysate may be useful.

## Data Availability

The datasets used during the current study are available from the corresponding author on reasonable request.

## Abbreviations

EP: Eosinophilic peritonitis
PD: Peritoneal dialysis
mNGS: Metagenomics next generation sequencing
WBC: White blood cell
CBC: Complete blood count
CKD-MBD: Chronic Kidney Disease–Mineral and Bone Disorder
CRP: C-reactive protein.
